# Epigenetic clock analysis of blood samples from Japanese schizophrenia patients

**DOI:** 10.1038/s41537-019-0072-1

**Published:** 2019-02-27

**Authors:** Satoshi Okazaki, Ikuo Otsuka, Shusuke Numata, Tadasu Horai, Kentaro Mouri, Shuken Boku, Tetsuro Ohmori, Ichiro Sora, Akitoyo Hishimoto

**Affiliations:** 1 Department of Psychiatry, Kobe University Graduate School of Medicine, Kobe, Japan; 2 Department of Psychiatry, Graduate School of Biomedical Sciences, Tokushima University, Tokushima, Japan; 3 Department of Internal Medicine, Division of Biosignal Pathophysiology, Kobe University Graduate School of Medicine, Kobe, Japan; 4 Medical Center for Student’s Health Service, Kobe University, Kobe, Japan

## Abstract

The accelerated aging hypothesis of schizophrenia (SCZ) has been proposed. DNA methylation profiles were developed for determining “epigenetic age.” Here, we assessed intrinsic and extrinsic epigenetic age acceleration (IEAA and EEAA, respectively) in SCZ. We examined two independent cohorts of Japanese ancestry. The first cohort consisted of 80 patients with SCZ under long-term or repeated hospitalization and 40 controls, with the economical DNA pooling technique. The second cohort consisted of 24 medication-free patients with SCZ and 23 controls. Blood of SCZ subjects exhibited decreased EEAA in the first cohort (*p* = 0.0162), but not in the second cohort. IEAA did not differ in either cohort. We performed replication analyses using publicly available datasets from European ancestry (three blood and one brain datasets). One blood dataset showed increased EEAA in SCZ (*p* = 0.0228). Overall, our results provide evidence for decreased EEAA in SCZ associated with hospitalization in the Japanese population.

## Introduction

Schizophrenia (SCZ) is a chronic and disabling psychiatric illness affecting ~1% of the general population.^1^ SCZ is associated with premature age-related phenotypes throughout the body. The brains of patients with SCZ exhibit old age-associated alterations, such as dendritic spine loss,^2,3^ cerebral cortical atrophy,^4^ and cognitive dysfunction.^5^ In addition, blood of patients with SCZ demonstrates premature age-related phenotypes, including telomere shortening,^6,7^ increased inflammatory markers,^8^ and elevated levels of oxidative stress,^9^ suggesting the involvement of multiple organ systems.

High mortality is observed in SCZ, resulting in a life expectancy of approximately 20 years below that of the general population.^10^ Most of the excess mortality has been attributed to natural causes such as cardiovascular and respiratory diseases, although mortality rates of unnatural causes such as suicide and accidental death are also higher in patients with SCZ.^11^ Both endogenous (such as genetic risk) and environmental (such as lifestyle and health care access) factors are suggested to contribute to premature mortality in SCZ. Tobacco smoking,^12^ sedentariness,^13^ obesity,^14^ insulin resistance,^15^ and hyperlipidemia,^16^ are all more common in patients with SCZ than in the general population. The accelerated aging hypothesis of SCZ has been advanced as a putative explanation for these observations. This hypothesis proposes that endogenous or environmental SCZ-associated factors accelerate the biological aging.^17,18^

Aging research has advanced substantially in recent years. López-Otín et al.^19^ enumerates nine candidate hallmarks of aging (epigenetic alterations, genomic instability, telomere attrition, loss of proteostasis, deregulated nutrient sensing, mitochondrial dysfunction, cellular senescence, stem cell exhaustion, and altered intercellular communication).^20^ Recently, independent research teams led by Horvath and Hannum^21,22^ developed two broadly accepted DNA methylation (DNAm) profiles for determining “epigenetic age” using 353 cytosine phosphate guanines (CpG) sites in multiple tissues and 71 CpG sites in blood samples, respectively. Epigenetic age acceleration on chronological age has been found to predict all-cause mortality.^23^ Further, Chen et al.^24^ expanded two refined measures of epigenetic age acceleration in blood samples: (a) intrinsic epigenetic age acceleration (IEAA) based on Horvath’s method, which is “pure” epigenetic aging independent of age-related changes in blood cell composition, and (b) extrinsic epigenetic age acceleration (EEAA), an enhanced version based on Hannum’s method, which up-weights the contribution of blood cell composition.

Epigenetic age acceleration is associated with various conditions, including neurological disorders such as Down syndrome,^25^ Alzheimer’s disease,^26^ Parkinson’s disease,^27^ bipolar disorder,^28^ and post-traumatic stress disorder.^29,30^ In SCZ, aberrant DNAm has been consistently reported,^31–33^ although several studies reported no accelerated epigenetic aging using blood or postmortem brain samples.^34–36^ However, these studies only examined universal epigenetic age acceleration as proposed by Horvath.

Here, we investigated the measures of epigenetic age acceleration, including IEAA and EEAA in blood samples of patients with SCZ and healthy controls, using two independent cohorts of Japanese ancestry. The first cohort consisted of 80 patients with SCZ under long-term or repeated hospitalization and 40 controls, with the economical DNA pooling technique.^37^ The second cohort consisted of 24 medication-free patients with SCZ and 23 controls, with a group of individuals. We found that blood of SCZ subjects exhibited decreased EEAA in the first cohort, but not in the second cohort. IEAA did not differ in either cohort. In addition, we performed replication analyses using independent publicly available DNA methylation datasets from populations of European ancestry (three blood and one brain datasets). One blood dataset showed increased EEAA in SCZ, in contrast with our data. Overall, our results provide evidence for decreased EEAA in SCZ associated with hospitalization in the Japanese population, and for the effect of environmental rather than endogenous factors to high mortality in SCZ.

## Results

### Accuracy of the epigenetic clock

**Fig. 1:**
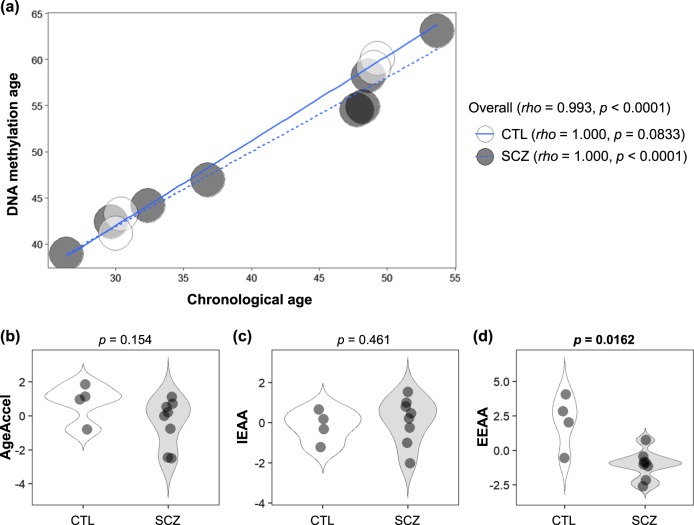
Epigenetic age acceleration analysis of the first cohort. **a** Scatter-plot of DNA methylation age vs. chronological age. Spearman’s correlation analysis indicates a significant positive correlation or trend toward positive correlation between chronological age and DNA methylation age in SCZ and control groups. **b**, **c**, **d** Violin-plots of measures of epigenetic age acceleration. Mann–Whitney *U*-test was performed for comparisons between SCZ and control groups. CTL, control; SCZ, schizophrenia; AgeAccel, universal epigenetic age acceleration; IEAA, intrinsic epigenetic age acceleration; EEAA, extrinsic epigenetic age acceleration

**Fig. 2:**
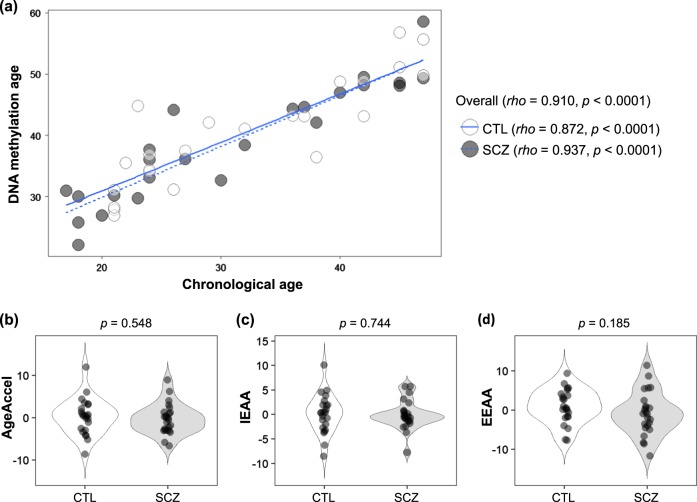
Epigenetic age acceleration analysis of the second cohort. **a** Scatter-plot of DNA methylation age vs. chronological age. Spearman’s correlation analysis indicates a significant positive correlation between chronological age and DNA methylation age in both SCZ and control groups. **b**, **c**, **d** Violin-plots of measures of epigenetic age acceleration. Mann–Whitney *U*-test was performed for comparisons between SCZ and control groups. CTL, control; SCZ, schizophrenia; AgeAccel, universal epigenetic age acceleration; IEAA, intrinsic epigenetic age acceleration; EEAA, extrinsic epigenetic age acceleration

DNAm age (also referred to as epigenetic age) was calculated as described by Horvath,^22^ from human samples profiled with the Illumina Infinium 450 K platform (450 K array). Demographic and clinical characteristics, as well as measures of epigenetic age acceleration are shown in Table 1. As expected, DNAm age was significantly correlated with chronological age in the first (rho = 0.993, *p* < 0.0001, Fig. 1) and second (rho = 0.910, *p* < 0.0001, Fig. 2) cohorts. The strong linear relationship between DNAm age and chronological age showed a high accuracy of the epigenetic clock was validated in both cohorts.

**Table 1:** Demographic and clinical characteristics, as well as measures of epigenetic age acceleration in the first and second cohorts

Cohort	First cohort *Japanese, Blood*			Second cohort *Japanese, Blood*		
	Control	Schizophrenia	*p*-value	Control	Schizophrenia	*p*-value
Characteristics						
Number	40 (4 pools)	80 (8 pools)		23	24	
Sex (male/female)	20/20	40/40	1.000^b^	10/13	11/13	0.871^b^
Age (years) (mean ± SD)	39.7 ± 11.6	40.5 ± 11.7	0.732^c^	31.9 ± 9.7	30.9 ± 10.5	0.726^c^
Duration of illness (years) (mean ± SD)	—	15.7 ± 11.3		—	—	
Antipsychotic dose^a^ (mg/day) (mean ± SD)	—	746 ± 732		—	Medication-free	
GAF score (mean ± SD)	—	34.0 ± 11.0		—	—	
BPRS score (mean ± SD)	—	54.4 ± 13.7		—	—	
Measures of epigenetic age acceleration^e^						
AgeAccel (years) (median [IQR])	1.053 (0.520, 1.318)	0.101 (−1.182, 0.579)	0.154^d^	0.499 (−2.743, 2.656)	−0.292 (−2.934, 1.258)	0.548^d^
IEAA (years) (median [IQR])	−0.071 (−0.548, 0.301)	0.339 (−0.448, 0.848)	0.461^d^	0.396 (−2.366, 1.932)	−0.502 (−1.363, 0.680)	0.744^d^
EEAA (years) (median [IQR])	2.430 (1.377, 3.147)	−0.957 (−1.398, −0.746)	**0.0162** ^d^	0.491 (−1.720, 3.931)	−0.930 (−4.149, 1.314)	0.185^d^

*AgeAccel* universal epigenetic age acceleration, *IEAA* intrinsic epigenetic age acceleration, *EEAA* extrinsic epigenetic age acceleration, *GAF* Global Assessment of Functioning, *BPRS* Brief Psychiatric Rating Scale, *SD* standard deviation, *IQR* interquartile range

Boldface type indicates significance

^a^Antipsychotic dose was calculated with chlorpromazine equivalents at blood draw

^b^*p*-value was calculated using *χ*^2^-test

^c^*p*-value was calculated using Student’s *t*-test

^d^*p-*value was calculated using Mann–Whitney *U*-test

^e^Measures of epigenetic age acceleration in the first cohort were calculated using pooled DNA samples

### Three measures of epigenetic age acceleration

We considered three measures of epigenetic age acceleration: universal epigenetic age acceleration (AgeAccel), IEAA, and EEAA, as detailed in Methods. In the first cohort, AgeAccel showed a trend toward a correlation with IEAA (rho = 0.566, *p* = 0.590) and EEAA (rho = 0.629, *p* = 0.0324). In the second cohort, AgeAccel showed a significant correlation with IEAA (rho = 0.797, *p* < 0.0001) and EEAA (rho = 0.480, *p* = 0.000735). On the other hand, there was no or a low correlation between IEAA and EEAA (rho = −0.0210, *p* = 0.956 in the first cohort; rho = 0.294, *p* = 0.0451 in the second cohort). By construction, the three measures of epigenetic age acceleration were uncorrelated with chronological age at the time of blood draw (rho = 0.0199—0.586, *p* = 0.169—0.894).

### Comparison of epigenetic age acceleration between SCZ and control

In the first cohort, we observed a significant difference between SCZ and control groups in EEAA (*p* = 0.0162), but not in AgeAccel (*p* = 0.154) or IEAA (*p* = 0.461) (Table 1 and Fig. 1). The median of EEAA in patients with SCZ and controls were −0.957 and 2.430 years, respectively; indicating that patients with SCZ had 3.387 years less EEAA than did controls. When stratified by age (cutoff of 35 years) or disease state (acute vs. chronic SCZ), there were no differences in each measure of epigenetic age acceleration (*p* > 0.05) (Supplementary Table 1a, b).

To address the confounding factor of antipsychotics in the patients, we subsequently performed a second investigation on medication-free patients (Table 1 and Fig. 2). In the second cohort, there was no significant difference in AgeAccel (*p* = 0.548), IEAA (*p* = 0.744), or EEAA (*p* = 0.185). These findings were inconsistent with those from the first cohort. When stratified by age, there were no differences in each measure of epigenetic age acceleration (*p* > 0.05) (Supplementary Table 1c).

### Replication analyses using publicly available DNA methylation datasets

**Fig. 3:**
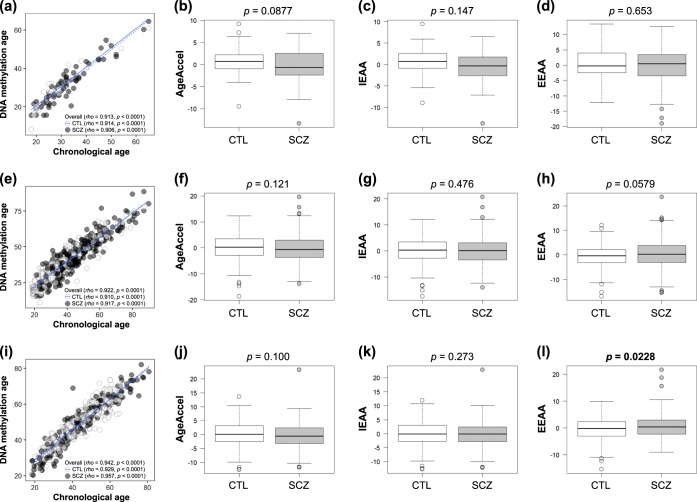
Epigenetic age acceleration analysis of the GSE41169, GSE80417, and GSE84727 datasets. **a**, **e**, **i** Scatter-plots of DNA methylation age vs. chronological age. Spearman’s correlation analysis indicates a significant positive correlation between chronological age and DNA methylation age in both SCZ and control groups. **b**, **c**, **d**, **f**, **g**, **h**, **j**, **k**, **l** Box-plots of measures of epigenetic age acceleration represent as follows: centerline, median; box limits, upper and lower quartile; whiskers, 1.5 × interquartile range; points, outliers. Mann–Whitney *U*-test was performed for comparisons between SCZ and control groups. CTL, control; SCZ, schizophrenia; AgeAccel, universal epigenetic age acceleration; IEAA, intrinsic epigenetic age acceleration; EEAA, extrinsic epigenetic age acceleration

The GSE41169, GSE80417, and GSE84727 datasets from blood for 450 K array were used for investigation of AgeAccel, IEAA, and EEAA between SCZ and control groups (Supplementary Table 2a and Fig. 3). In the GSE41169 and GSE80417 datasets, there were no significant differences in the three measures of epigenetic age acceleration (*p* > 0.05). In the GSE84727 dataset, we observed a significant difference in EEAA (*p* = 0.0228), but not in AgeAccel or IEAA (*p* > 0.05). The median of EEAA in patients with SCZ and controls were 0.377 and −0.233 years, respectively, indicating that patients with SCZ had 0.610 years more EEAA than did controls.

Furthermore, the GSE38873 dataset from postmortem cerebellum brain for the Illumina Infinium 27 K platform (27 K array) array was used to investigate AgeAccel among SCZ, bipolar disorder (BPD), major depressive disorder (MDD), and control groups (Supplementary Table 2b and Supplementary Fig. 1). As a result, there was no significant difference among them (*p* > 0.05).

## Discussion

We investigated measures of epigenetic age acceleration, including IEAA and EEAA in SCZ. DNAm age was strongly correlated with chronological age in pooled DNA samples. The economical DNA pooling technique generates accurate quantitative assessments to detect group differences in DNA methylation averages; this reduces the time, cost, and amount of DNA starting material required. It will prove valuable in highlighting candidate regions of the genome, which warrant further large-scale studies involving multiple regions at the individual-sample level.^37^ In addition, the present study demonstrated that the method is useful for measuring epigenetic age acceleration when funds and DNA stocks are limited.

Universal epigenetic age acceleration (AgeAccel) in blood did not differ between SCZ and control groups in both first and second cohorts, consistent with a previous study using European blood DNAm datasets.^38^ IEAA also did not differ in both cohorts. In contrast, EEAA in patients with SCZ was significantly decreased compared to that of controls in the first cohort, although no significant reduction was observed in the second cohort. We proposed several reasons for this. First, the sample size of the second cohort was insufficient (sample power = 0.216). Second, a detectable difference in EEAA between cases and controls may require a certain time course. The mean ages of the first and second cohorts differed by a decade (approximately 40 and 30 years old, respectively), which may have obscured differences in EEAA.

Next, we performed replication analyses with publicly available DNAm datasets from European blood (GSE41169, GSE80417, and GSE84727) and brain (GSE38873) samples. The GSE80417 and GSE84727 datasets were used in McKinney et al.,^35^ but they did not use the IEAA or EEAA method. As a result, EEAA in SCZ was significantly increased compared to that of controls in the GSE84727 dataset, which differed from our data. The comparison of AgeAccel in brain among SCZ and other psychiatric diseases using the GSE38873 dataset showed no significant difference.

These findings were in contrast to the accelerated aging hypothesis of SCZ. Taken together, AgeAccel and IEAA did not differ between SCZ and control groups. In fact, median values of AgeAccel for SCZ groups were lower than that for control groups in all blood datasets studied here, and similar tendencies were shown in previous studies.^34–36^ However, no definitive conclusion could be drawn, because there was no statistically significant difference in any comparison. On another front, EEAA showed heterogeneous results among our data and the publicly available datasets. The findings from our first cohort suggested that decreased EEAA in patients with SCZ in the Japanese population, although those from the GSE84727 dataset presented contradictory results in European populations.

EEAA captures age-related changes in leukocyte composition, is correlated with lifestyle and health-span related characteristics, and yields a stronger predictor of all-cause mortality; whereas IEAA measures cell-intrinsic methylation changes unrelated with lifestyle factors.^24,39^ Both EEAA and IEAA relate to indicators of metabolic syndrome (MetS).^39^ It is well known that SCZ-associated factors (such as tobacco smoking, sedentariness, obesity, insulin resistance, and hyperlipidemia) and antipsychotic medication can lead to MetS. Many studies have reported a higher MetS prevalence in patients with SCZ than in the general population.^40,41^ Meanwhile, hospitalization seems to contribute to a better lifestyle, due to restricted smoking and alcohol use, regular diet, and appropriate exercise. A Japanese nationwide large-scale research showed that MetS prevalence in inpatients with SCZ (13.0%) was lower than in outpatients (34.2%) or in the general population (15.6%).^42^ In Japan, the mental health care system remains hospital-based, and has the largest number of psychiatric beds (2.8 beds per 1000 persons) and the longest average length of hospital stay of psychiatric inpatients (approximately 300 days) in the world.^43^ Indeed, the patients with SCZ in the first cohort were mainly under long-term or repeated hospitalization. In addition, significantly longer leukocyte telomere length, one of the candidate hallmarks of aging described above, was observed in elderly patients with SCZ under long-term hospitalization in Japan.^44^ Considering these findings, we posit that health characteristics in patients with SCZ under hospitalization could delay EEAA, and that SCZ itself does not have robust effects on epigenetic age acceleration. Furthermore, high mortality in SCZ seems to be the effect of environmental rather than endogenous SCZ-associated factors, and may be improved by appropriate lifestyle management.

There are several limitations to the present study. First, the sample size of our cohorts was relatively small and had a narrow age range. That seems to be the reason why overall samples exhibited differences between DNAm age and chronological age in our cohorts and the GSE38873 dataset. Further studies employing larger samples with broader age range will be required, especially considering the effects of medication, hospitalization, and age. Second, our results may not be generalizable beyond the Japanese population, and cannot be extrapolated as universal changes of epigenetic aging in SCZ. Further comparisons with different populations are required. Third, the measures of epigenetic age acceleration were only examined in blood or cerebellum brain samples. Thus, we cannot rule out the possibility of accelerated epigenetic age that may be present in other tissues or defined cell types, including different brain regions or neural/glial cells. Fourth, we could not obtain detailed information regarding several potential confounders that are known to affect epigenetic aging, including alcohol- and tobacco-abuse, education, Mets, and associated pathologies.^39,45^

In conclusion, we provide evidence for decreased EEAA in patients with SCZ under long-term or repeated hospitalization in the Japanese population. In addition, these findings suggest the effect of environmental rather than endogenous SCZ-associated factors to high mortality in SCZ. Further studies using larger samples, different populations, or other tissues and defined cell types will be necessary to elucidate epigenetic aging mechanisms in SCZ.

## Methods

### Participants

This study was conducted in accordance with the Declaration of Helsinki and was approved by the institutional ethics committees of Kobe University Graduate School of Medicine and the University of Tokushima Graduate School. After receiving a complete description of the study, written informed consent was obtained from all participants.

Two independent cohorts were examined. Demographic and clinical characteristics are shown in Table 1. The first cohort consists of 80 patients with SCZ (40 men and 40 women; mean age ± SD, 40.5 ± 11.7 years) and 40 healthy controls (20 men and 20 women; 39.7 ± 11.6 years), who were recruited from Kobe University.^46^ These patients were mainly under long-term or repeated hospitalization. The second cohort consists of 24 medication-free patients with SCZ (11 men and 13 women; 30.9 ± 10.5 years) and 23 healthy controls (10 men and 13 women; 31.9 ± 9.7 years), who were recruited from Tokushima University.^31^ Among the patients, 16 patients had no history of taking antipsychotics; of the other eight patients, seven had not taken any antipsychotics for at least 2 months.

Psychiatric assessments of participants were performed as previously described.^47^ A diagnosis of SCZ was given by at least two psychiatrists according to the DSM-IV criteria for SCZ based on unstructured interviews and reviews of their medical records. Control participants were healthy volunteers, screened for psychiatric disorders by a psychiatrist. None had any present, past, or family (first-degree relatives) history of psychiatric disorders or substance abuse, excluding nicotine dependence.

### Publicly available DNA methylation datasets

Replication analyses were performed using independent DNAm datasets downloaded from the Gene Expression Omnibus database (Supplementary Table 2a and 2b). The GSE41169, GSE80417, and GSE84727 datasets are blood DNAm data using 450 K array. The GSE41169 dataset was generated by Horvath et al.,^48^ consisting of 62 patients with SCZ and 33 controls from the University Medical Center Utrecht in Netherland. The GSE80417 dataset was generated by Hannon et al.,^49^ consisting of subjects from University College London in England. After excluding subjects with missing or errant chronological age information as described in McKinney et al.,^35^ analysis was performed on 332 patients with SCZ and 304 controls. The GSE84727 dataset was generated by Hannon et al.,^49^ consisting of subjects from the University of Aberdeen in Scotland.^50^ After excluding subjects with missing chronological age information as described in McKinney et al.,^35^ analysis was performed on 260 patients with SCZ and 405 controls. The GSE38873 dataset is postmortem cerebellum brain DNAm data using 27 K array, and was generated by Chen et al.,^51^ consisting of subjects from the Stanley Medical Research Institute Neuropathology Consortium and Array collection.^52^ After excluding replicates, analysis was performed on 45 patients with SCZ, 46 patients with BPD, 15 patients with MDD, and 47 controls.

### Calculation of DNAm age and aging acceleration

Genomic DNA extractions from our peripheral blood samples and measures of DNAm were conducted as described previously.^31,46^ In the first cohort, equal amounts of DNA (20 ng) for each sample were pooled from ten individuals in each group. Thus, we prepared 12 pooling DNA samples from 120 individuals and performed comparisons among the three groups (four acute SCZ pools vs. four chronic SCZ pools vs. four healthy control pools) (Supplementary Table 3). Genome-wide DNAm was investigated using the Illumina Infinium HumanMethylation450 BeadChip (Illumina, San Diego, CA).

DNAm-based age prediction and measures of epigenetic age acceleration were implemented by the online DNAm age calculator (https://horvath.genetics.ucla.edu/html/dnamage/).^22^ DNAm age was calculated as described by Horvath.^22^ We considered measures of epigenetic age acceleration consistent with other publications. The universal measure of age acceleration (AgeAccel) is defined as the residual from regressing DNAm age on chronological age, and is denoted as *AgeAccelerationResidual* in the online calculator. A positive (or negative) value indicates that the biological age of the sample is higher (or lower) than expected based on chronological age. Next, we considered IEAA and EEAA, which only apply to blood data using 450 K array. IEAA indicates “pure” epigenetic aging effects in blood that are not confounded by differences in blood cell counts. EEAA indicates aging in immune-related components also associated with age-related changes in blood cell counts.^24,27^ IEAA and EEAA are denoted as *AAHOAdjCellCounts* and *BioAge4HAStaticAdjAge* in the online calculator, respectively.^39^ We performed the correlation analyses to examine the independency of the three measures, especially IEAA and EEAA, in our cohorts.

### Statistics

Statistical analyses were performed using R version 3.4.1 (The R Foundation for Statistical Computing, Vienna, Austria) with EZR version 1.37 (Jichi Medical University, Saitama, Japan).^53^ The differences in continuous variables between groups were analyzed with Mann–Whitney *U*-test or Kruskal–Wallis rank sum test. The relationship between continuous variables was analyzed with Spearman’s rank correlation coefficient. Power analysis was performed with G*Power version 3.1.9.2 (Universität Kiel, Kiel, Germany).^54^ Statistical significance was defined as two-tailed *p* < 0.05.

### Reporting summary

Further information on experimental design is available in the Nature Research Reporting Summary linked to this article.

## Supplementary information

Reporting Summary

Supplementary Information.

## Data Availability

The publicly available datasets were downloaded from the Gene Expression Omnibus database as follows: GSE41169,^48^ GSE80417,^49^ GSE84727,^49^ GSE38873.^51^ The other data supporting the findings of this study are available from the corresponding author upon reasonable request.

## Supplementary information

**Supplementary information** accompanies the paper on the *npj Schizophrenia* website (10.1038/s41537-019-0072-1).
