# Supplementary material for: Epigenetic clock analysis of blood samples from Japanese schizophrenia patients
Source: NPJ Schizophr. 2019 Feb 27;5:4. doi: 10.1038/s41537-019-0072-1 (PMC6393510; doi:10.1038/s41537-019-0072-1)
Supplement: Supplementary file 2 — Supplementary Information. [file 41537_2019_72_MOESM2_ESM.pdf]

Supplementary Fig. 1. Epigenetic age acceleration analysis of the GSE38873 dataset.

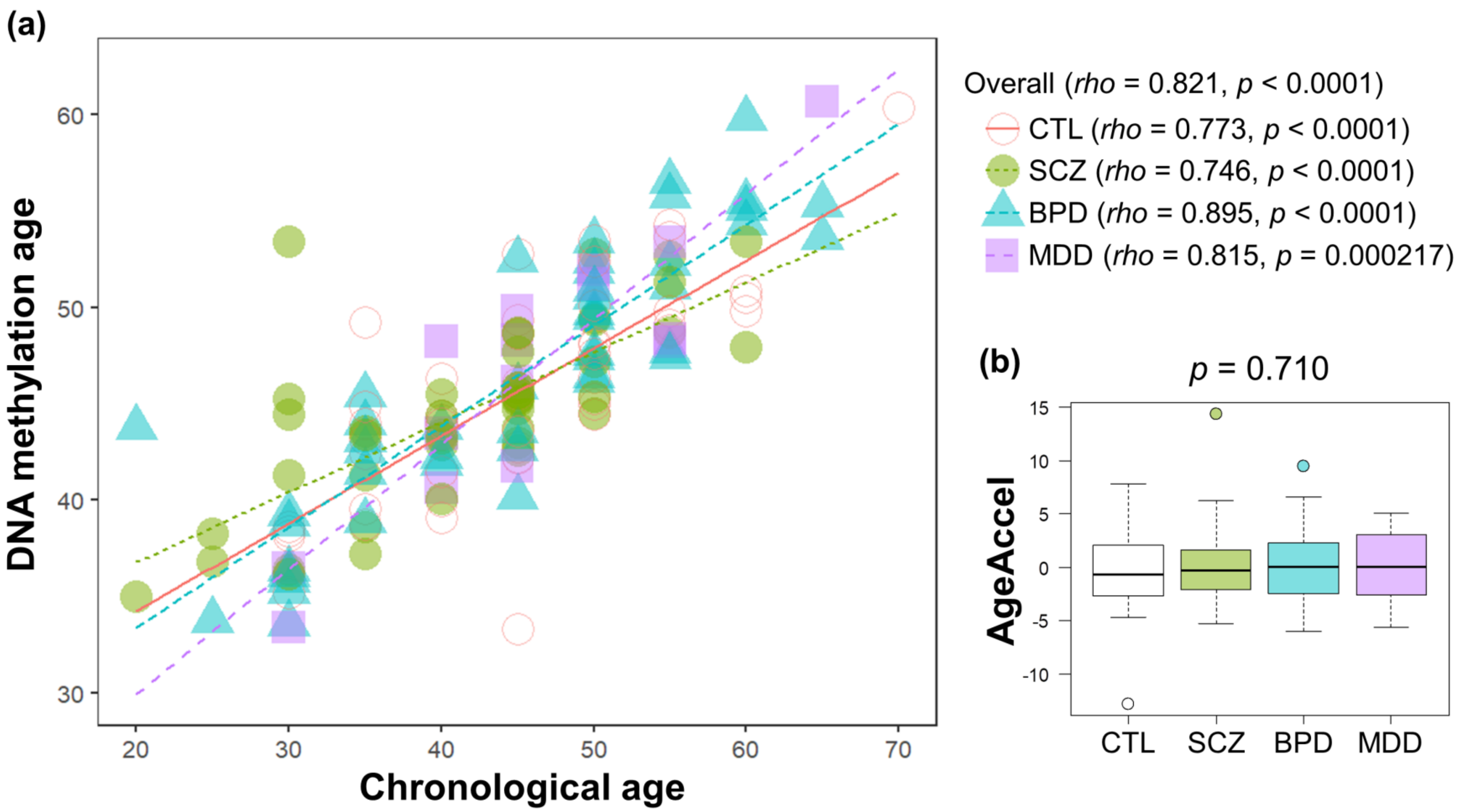

(**a**) Scatter-plot of DNA methylation age vs. chronological age. Spearman's correlation analysis indicates a significant positive correlation between chronological age and DNA methylation age in each group. (**b**) Box-plots of universal epigenetic age acceleration represent as follows: centerline, median; box limits, upper and lower quartile; whiskers, 1.5× interquartile range; points, outliers. Kruskal-Wallis rank sum test was performed for comparisons among SCZ, BPD, MDD, and control groups.

CTL, control; SCZ, schizophrenia; BPD, bipolar disorder; MDD, major depressive disorder; AgeAccel, universal epigenetic age acceleration.

Supplementary Table 1a. Comparison of measures of epigenetic age acceleration between acute and chronic schizophrenia in the first cohort.

	Acute SCZ	Chronic SCZ	*P*-value
**Characteristics**			
Number	40 (4 pools)	40 (4 pools)	
Sex (male/female)	20 / 20	20 / 20	1.000[b]
Age (yeas) (mean ± SD)	41.3 ± 12.9	39.6 ± 10.4	0.531[c]
Duration of illness (years) (mean ± SD)	16.0 ± 12.2	15.3 ± 10.4	0.804[c]
Antipsychotic dose[a] (mg/day) (mean ± SD)	565 ± 710	927 ± 717	0.0263[c]
GAF score (mean ± SD)	27.2 ± 7.8	40.7 ± 9.6	< 0.001[c]
BPRS score (mean ± SD)	63.4 ± 11.7	45.5 ± 9.0	< 0.001[c]
**Measures of epigenetic age acceleration***			
AgeAccel (years) (median [IQR])	−0.379 (−1.182, 0.278)	0.366 (−0.473, 0.579)	0.886[d]
IEAA (years) (median [IQR])	0.101 (−0.448, 0.594)	0.508 (−0.344, 0.984)	0.886[d]
EEAA (years) (median [IQR])	−1.537 (−2.282, −0.488)	−0.931 (−1.046, −0.746)	0.686[d]

SCZ, schizophrenia; GAF, Global Assessment of Functioning; BPRS, Brief Psychiatric Rating Scale; SD, standard deviation; AgeAccel, universal epigenetic age acceleration; IEAA, intrinsic epigenetic age acceleration; EEAA, extrinsic epigenetic age acceleration; IQR, interquartile range.

* Measures of epigenetic age acceleration were calculated using pooled DNA samples.

[a] Antipsychotic dose was calculated with chlorpromazine equivalents at blood draw.

[b] *P*-value was calculated using χ^2-test.

[c] *P*-value was calculated using Student's t-test.

[d] *P*-value was calculated using Mann-Whitney U-test.

Supplementary Table 1b. Comparison of measures of epigenetic age acceleration between younger and older schizophrenia in the first cohort.

	Younger SCZ	Older SCZ	*P*-value
**Characteristics**			
Number	40 (4 pools)	40 (4 pools)	
Sex (male/female)	20 / 20	20 / 20	1.000[b]
Age (yeas) (mean ± SD)	31.3 ± 6.8	49.6 ± 7.7	< 0.001[c]
Duration of illness (years) (mean ± SD)	10.0 ± 6.0	21.3 ± 12.5	< 0.001[c]
Antipsychotic dose[a] (mg/day) (mean ± SD)	697 ± 662	796 ± 801	0.549[c]
GAF score (mean ± SD)	32.6 ± 11.7	35.3 ± 10.2	0.276[c]
BPRS score (mean ± SD)	54.9 ± 13.9	53.9 ± 13.7	0.747[c]
**Measures of epigenetic age acceleration***			
AgeAccel (years) (median [IQR])	0.101 (−0.189, 0.331)	−0.959 (−2.461, 0.676)	0.886[d]
IEAA (years) (median [IQR])	0.339 (0.096, 0.730)	−0.104 (−1.263, 0.848)	0.686[d]
EEAA (years) (median [IQR])	−0.872 (−1.217, −0.746)	−1.077 (−1.505, -0.576)	0.686[d]

SCZ, schizophrenia; GAF, Global Assessment of Functioning; BPRS, Brief Psychiatric Rating Scale; SD, standard deviation; AgeAccel, universal epigenetic age acceleration; IEAA, intrinsic epigenetic age acceleration; EEAA, extrinsic epigenetic age acceleration; IQR, interquartile range.

* Measures of epigenetic age acceleration were calculated using pooled DNA samples.

[a] Antipsychotic dose was calculated with chlorpromazine equivalents at blood draw.

[b] *P*-value was calculated using χ^2-test.

[c] *P*-value was calculated using Student's t-test.

[d] *P*-value was calculated using Mann-Whitney U-test.

Supplementary Table 1c. Comparison of measures of epigenetic age acceleration between younger and older schizophrenia in the second cohort.

	Younger SCZ (age < 35)	Older SCZ (age ≥ 35)	*P*-value
**Characteristics**			
Number	14	10	
Sex (male/female)	10 / 4	1 / 9	0.0029[a]
Age (yeas) (mean ± SD)	23.0 ± 4.7	41.9 ± 4.1	< 0 .001[b]
**Measures of epigenetic age acceleration**			
AgeAccel (years) (median [IQR])	−0.756 (−3.058, 2.143)	0.103 (−2.526, 0.852)	0.752[c]
IEAA (years) (median [IQR])	−0.502 (−1.094, 0.631)	−0.407 (−1.402, 0.595)	1.000[c]
EEAA (years) (median [IQR])	−0.738 (−4.630, 0.851)	−1.694 (−3.836, 4.210)	0.977[c]

SCZ, schizophrenia; SD, standard deviation; AgeAccel, universal epigenetic age acceleration; IEAA, intrinsic epigenetic age acceleration; EEAA, extrinsic epigenetic age acceleration; IQR, interquartile range.

[a] *P*-value was calculated using χ^2-test.

[b] *P*-value was calculated using Student's t-test.

[c] *P*-value was calculated using Mann-Whitney U-test.

Supplementary Table 2a. Demographic and clinical characteristics, as well as measures of epigenetic age acceleration in the publicly-available blood DNA methylation datasets.

**Cohort**	**GSE41169** *Dutch, Blood*			**GSE80417** *White British, Blood*			**GSE84727** *White British, Blood*		
	Control	Schizophrenia	*P*-value	Control	Schizophrenia	*P*-value	Control	Schizophrenia	*P*-value
**Characteristics**									
Number	33	62		304	332		405	260	
Sex (male/female)	21 / 12	46 / 16	0.283[a]	135 / 169	242 / 90	< 0.001[a]	303 / 102	177 / 83	0.0585[a]
Age (yeas) (mean ± SD)	29.4 ± 10.3	32.7 ± 10.2	0.128[b]	36.8 ± 14.7	43.7 ± 14.6	< 0.001[b]	44.9 ± 12.2	44.2 ± 14.1	0.515[b]
**Measures of epigenetic age acceleration**									
AgeAccel (years) (median [IQR])	0.714 (−0.963, 2.181)	−0.670 (−2.351, 2.505)	0.0877[c]	0.288 (−2.900, 3.448)	−0.691 (−3.649, 2.996)	0.121[c]	0.059 (−2.515, 3.126)	−0.550 (−3.176, 2.344)	0.100[c]
IEAA (years) (median [IQR])	0.745 (−0.854, 2.648)	−0.306 (−2.619, 1.730)	0.147[c]	0.287 (−2.743, 3.473)	0.129 (−3.396, 3.100)	0.476[c]	−0.061 (−2.763, 2.986)	−0.068 (−2.842, 2.445)	0.273[c]
EEAA (years) (median [IQR])	−0.302 (−2.532, 3.927)	0.407 (−3.430, 3.385)	0.653[c]	−0.360 (−3.189, 2.205)	0.272 (−3.031, 3.823)	0.0579[c]	−0.233 (−3.096, 2.362)	0.377 (−2.226, 2.921)	**0.0228**[c]

AgeAccel, universal epigenetic age acceleration; IEAA, intrinsic epigenetic age acceleration; EEAA, extrinsic epigenetic age acceleration; SD, standard deviation; IQR, interquartile range.

[a] *P*-value was calculated using χ^2-test.

[b] *P*-value was calculated using Student's t-test.

[c] *P*-value was calculated using Mann-Whitney U-test.

Supplementary Table 2b. Demographic and clinical characteristics, as well as measures of epigenetic age acceleration of younger samples in the publicly-available brain DNA methylation dataset.

Cohort	**GSE38873** *European, Brain*				
	CTL	SCZ	BPD	MDD	*P*-value
**Characteristics**					
Number	47	45	46	15	
Age (yeas) (mean ± SD)	45.1 ± 9.1	41.8 ± 9.4	44.8 ± 11.4	46.0 ± 9.5	0.302[a]
PMI (hours) (mean ± SD)	28.2 ± 12.4	32.8 ± 15.6	35.9 ± 17.6	27.5 ± 10.7	0.0559[a]
pH (mean ± SD)	6.51 ± 0.29	6.39 ± 0.28	6.36 ± 0.30	6.18 ± 0.21	0.00115[a]
**Measures of epigenetic age acceleration**					
AgeAccel (years) (median [IQR])	−0.638 (−2.666, 2.102)	−0.290 (−2.128, 1.622)	0.050 (−2.409, 2.206)	0.027 (−2.579, 3.096)	0.710[b]

CTL, control; SCZ, schizophrenia; BPD, bipolar disorder; MDD, major depressive disorder; AgeAccel, universal epigenetic age acceleration; PMI, postmortem interval; SD, standard deviation; IQR, interquartile range.

Sex information was not listed in the dataset.

[a] *P*-value was calculated using one-way ANOVA.

[b] *P*-value was calculated using Kruskal-Wallis rank sum test.

Supplementary Table 3. Demographic and clinical characteristics, as well as measures of epigenetic age acceleration of DNA pools in the first cohort

Pooled sample number	1	2	3	4	5	6	7	8	9	10	11	12
**Characteristics**												
Phenotype	CTL	CTL	CTL	CTL	aSCZ	aSCZ	aSCZ	aSCZ	cSCZ	cSCZ	cSCZ	cSCZ
Sex	male	male	female	female	male	male	female	female	male	male	female	female
Age (yeas)	30.4	49.3	30.0	49.0	26.4	48.2	36.8	53.7	29.7	48.6	32.4	47.8
Duration of illness (years)	-	-	-	-	7.3	23.1	12.5	21.0	9.1	21.3	11.2	19.9
Antipsychotic dose[a] (mg/day)	-	-	-	-	730	555	340	638	871	1189	846	802
GAF score	-	-	-	-	27.1	28.7	23.5	29.6	38.1	40.1	41.8	42.9
BPRS score	-	-	-	-	62.7	64.7	65.1	61.0	49.0	47.3	42.9	42.7
**Measures of epigenetic age acceleration**												
DNAm age (years)	26.6	45.5	35.8	55.5	30.5	48.6	32.6	45.5	31.6	51.8	29.0	49.4
AgeAccel (years)	0.217	−2.747	−1.004	1.807	0.759	0.006	0.192	−2.254	1.196	2.457	−0.979	0.352
IEAA (years)	0.475	−1.168	0.088	0.875	0.272	0.013	1.320	−1.157	−0.841	0.484	−0.882	0.521
EEAA (years)	−2.489	−2.505	−0.979	0.410	−0.606	−1.017	−0.521	−0.647	2.898	3.891	2.153	−0.590

CTL, control; aSCZ, acute schizophrenia; cSCZ, chronic schizophrenia; GAF, Global Assessment of Functioning; BPRS, Brief Psychiatric Rating Scale; DNAm age, DNA methylation age; AgeAccel, universal epigenetic age acceleration; IEAA, intrinsic epigenetic age acceleration; EEAA, extrinsic epigenetic age acceleration

We prepared 12 pooled DNA samples from 120 individuals. In each group (control, acute schizophrenia, and chronic schizophrenia), 40 individuals were divided into 4 pools (10 younger males, 10 older males, 10 younger females, and 10 older females). Data for continuous variables of characteristics are presented as the average in each pool.

[a] Antipsychotic dose was calculated with chlorpromazine equivalents at blood draw.
